# On the stoichiometry and stability of americium(III) complexes with a hydrophilic SO_3_–Ph–BTP ligand, studied by liquid–liquid extraction

**DOI:** 10.1007/s10967-015-4663-7

**Published:** 2015-12-26

**Authors:** Łukasz Steczek, Magdalena Rejnis, Jerzy Narbutt, Marie-Christine Charbonnel, Philippe Moisy

**Affiliations:** Department – Centre for Radiochemistry and Nuclear Chemistry, Institute of Nuclear Chemistry and Technology, Dorodna 16, 03-195 Warsaw, Poland; Nuclear Energy Division, RadioChemistry & Processes Department, CEA Marcoule, 30207 Bagnols sur Cèze, France

**Keywords:** Americium(III), Complexes, Hydrophilic BTP ligand, Partitioning, Solvent extraction

## Abstract

1:1 and 1:2 complexes of americium(III) with a hydrophilic anionic SO_3_–Ph–BTP^4−^ ligand were detected in acidic aqueous nitrate solutions by a solvent extraction method. The determined conditional stability constants of these complexes, log*β*_1_ = 4.35 ± 0.07 and log*β*_2_ = 7.67 ± 0.06, related to 1 M aqueous solutions, are much lower than the literature values for the analogous curium species, determined by TRLFS in very dilute aqueous solutions. There is also no evidence for the existence of the 1:3 Am^3+^ complex similar to the reported curium(III) complex. A hypothesis has been formulated to explain these discrepancies. It suggests the necessity to carefully check the equilibria in each phase of solvent extraction systems containing two competing ligands—lipophilic and hydrophilic.

## Introduction

**Scheme 1:**
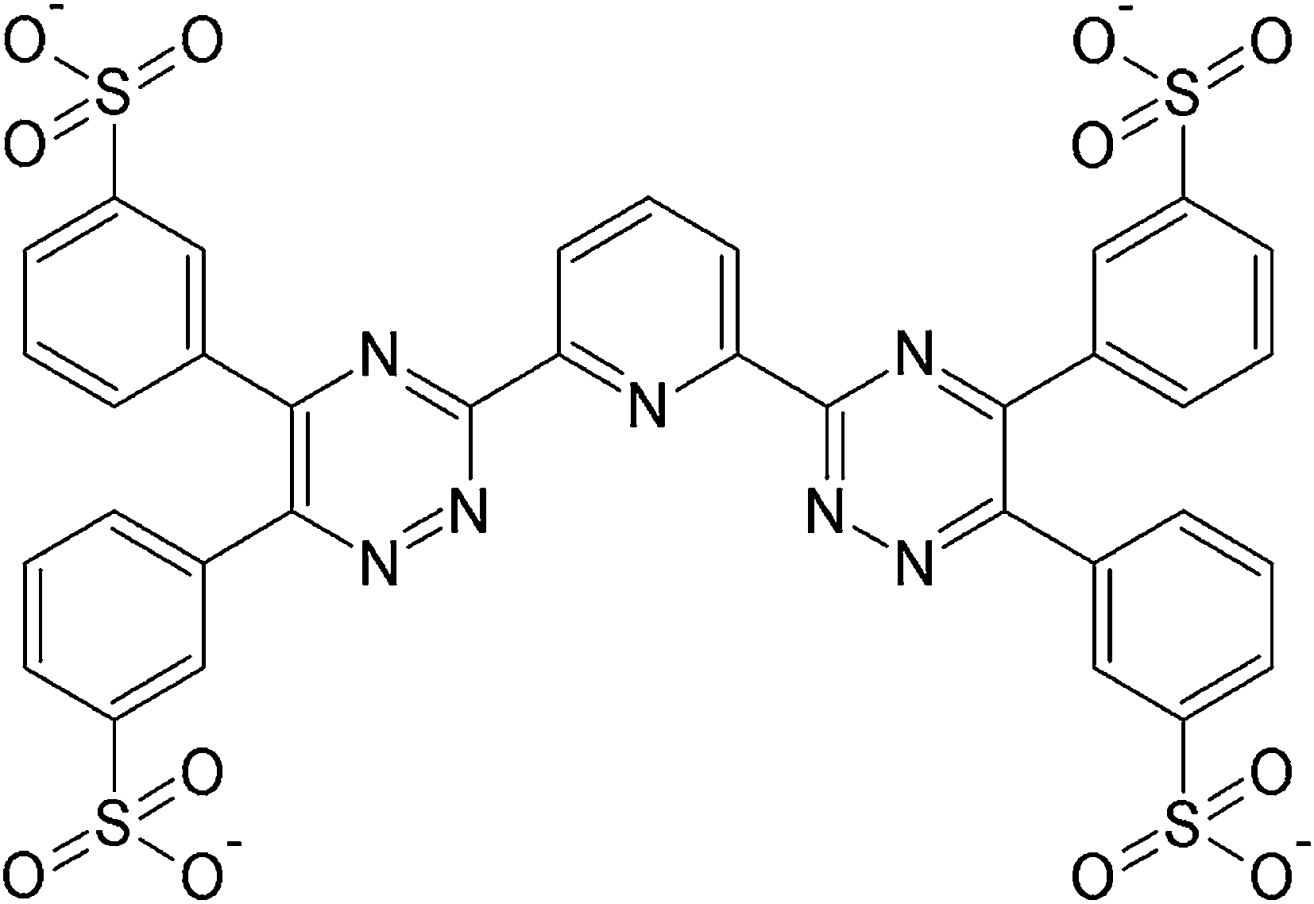
Structural formula of the SO_3_–Ph–BTP^4−^ anion. Reprinted from Ref. [18] with the permission from the Editor of Nukleonika

Removal of minor actinides (MA) from nuclear waste, in particular separation of Am(III) from the lanthanide fission products (Ln), is a necessary step in the strategy of partitioning and transmutation (P&T) [1]. Lipophilic poly-N-dentate ligands, derivatives of bis-triazinyl-pyridine (BTP), bis-triazinyl-bipyridine (BTBP) and bis-triazinyl-phenantroline (BTPhen) are highly effective extractants for the separation of trivalent MA (americium, curium) from lanthanides in HNO_3_ solutions [2, 3]. Another solvent extraction approach to separate MA from Ln implies the use of actinide-selective hydrophilic ligands, as in the processes of innovative Selective Actinide Extraction (i-SANEX) or Group Actinide Extraction (GANEX) [2]. Such an approach was already considered in the reverse TALSPEAK process, where water-soluble aminopolycarboxylate complexants in buffered media were proposed to selectively strip the actinides from the MA/Ln loaded organic phase [4]. However, the necessity of rigid pH control within a narrow range of pH ≥3, required for the polyaminocarboxylates, is undesirable for an industrial process. Novel hydrophilic complexants have been developed, operating in more acidic media. The most efficient actinide-selective agent proposed for MA stripping to acidic aqueous solutions is a hydrophilic, anionic BTP ligand, 2,6-bis(5,6-di(sulfophenyl)-1,2,4-triazin-3-yl)pyridine (SO_3_–Ph–BTP—Scheme 1), developed for the i-SANEX process [2, 5, 6]. Also other similar sulfonated poly-N-dentate hydrophilic ligands were studied as potential Am(III) stripping agents [7, 8]. Still other hydrophilic chelating ligands proposed for selective MA stripping—completely incinerable ‘CHON’ molecules, for example neutral N,O-bitopic derivatives of 1,10-phenantroline [9] and other similar chelators [10, 11], as well as cationic quaternary ammonium derivatives of tetra-N-dentate BTBP [Lewis FW et al. in preparation]—are less efficient in separating MA from Ln.

In such processes the An/Ln separation occurs after a first step where the actinides have been loaded into an organic phase containing a non-selective lipophilic An/Ln extractant, for example *N,N,N′,N*′-tetraoctyl-diglycolamide (TODGA) [12, 13]. Because the values of stability constants (log*β*_3_) of Cm^3+^ complexes with a neutral lipophilic nPr-BTP ligand [14] are higher than those of the TODGA complexes [15] (in similar alcohol-water solvents), we may conclude that the negatively charged SO_3_–Ph–BTP^4−^ ligand would form still stronger Am^3+^ complexes than the neutral TODGA (chemistry of Cm^3+^ is very similar to that of Am^3+^). No stability constants of the Am^3+^—SO_3_–Ph–BTP complexes have been found in literature, but such data are available for Cm^3+^ for which the time-resolved laser fluorescence spectroscopy (TRLFS) technique can be used. Stability constants of the 1:1. 1:2 and 1:3 Cm^3+^—SO_3_–Ph–BTP^4−^ complexes in aqueous solutions have been reported by Geist et al. [16]. However, solvent extraction studies by the same team, carried out with the system TODGA/SO_3_–Ph–BTP + HNO_3_, seem to suggest the presence of only two (1:1 and 1:2) Am^3+^—SO_3_–Ph–BTP complexes in the aqueous phase [5]. In spite of extensive studies focused on the An(III)/Ln(III) complexation by SO_3_–Ph–BTP, the reason of the above discrepancy has not been explained yet.

The knowledge of complexing properties (in respect to the actinides and lanthanides) of ligands used in solvent extraction processes is of paramount importance for designing novel separation schemes. The aim of the present work was to determine the stability constants and to contribute to the understanding of the peculiar complexation of Am(III) by SO_3_–Ph–BTP in the solvent extraction system studied. In order to determine the number and stoichiometries of the americium(III)—SO_3_–Ph–BTP^4−^ complexes in the acidic (HNO_3_) aqueous phase, and to calculate their stability constants we analysed the variation of the Am^3+^ distribution ratio between the TODGA organic phase and the SO_3_–Ph–BTP/HNO_3_ aqueous phase as a function of the concentration of the hydrophilic ligand. By taking into account the formation of an extractable metal complex with the lipophilic ligand and the formation of the metal complexes with the hydrophilic ligand in the aqueous phase, the stability constants of the latter could be obtained [17].

## Model of the solvent extraction process

In our recent work on the complexation of uranyl(VI) cation with SO_3_–Ph–BTP^4−^ (L^4−^), using the same solvent extraction technique, we presented a model of solvent extraction process in the system containing two competing ligands: neutral lipophilic TODGA and anionic hydrophilic SO_3_–Ph–BTP^4−^ (L^4−^) [18]. The same model for the case of Am^3+^ ions is given below:

1 $${\text{Am}}^{ 3+ } + \, i{\text{TODGA}}_{\text{org}} + {\text{ 3NO}}_{3}^{ - } \mathop{\rightleftharpoons}\limits^{K_{ex} } {\text{Am}}\left( {\text{TODGA}} \right)_{i} \left( {{\text{NO}}_{ 3} } \right)_{{ 3,{\text{org}}}}$$

2 $${\text{Am}}^{ 3+ } + \, i{\text{L}}^{{ 4{-}}} \mathop{\rightleftharpoons}\limits^{K_{{{\text{L,}}i}}} {\text{AmL}}_{i}^{{ 3{-} 4i}}$$

3 $${\text{Am}}^{ 3+ } + j{\text{NO}}_{3}^{ - } \mathop{\rightleftharpoons}\limits^{K_{{{\text{NO}}_{ 3} ,j}} } {\text{Am(NO}_{3})_{j}^{3- j}}$$

4 $${\text{L}}^{{ 4{-}}} + {\text{iH}}^{ + }\mathop{\rightleftharpoons}\limits^{K_{\rm H,i}} {\text{H}}_{\text{i}} {\text{L}}^{{\rm i} - 4}$$

where subscript “org” denotes the species in the organic phase, and the lack of subscript—the species in the aqueous phase.

The experiments were performed at rather high acidities (pH <2) and at a constant ionic strength (1 M ${\text{NO}}_{3}^{ - }$). We can expect that under these conditions: (i) Am^3+^ ions are not hydrolyzed; (ii) HNO_3_ is nearly totally dissociated; (iii) the Na^+^ ions present in the aqueous phase do not interact with the L^4−^ ligand; and (iv) the equilibrium constants (Eqs. 5–8) are the conditional constants related to I = 1 M. Solubility of TODGA in the aqueous phase is negligible [19], as well as that of SO_3_–Ph–BTP in the organic phase [18]. From Eqs. (1–4) we obtain:

5 $$K_{\text{ex}} = \frac{{ [ {\text{Am(TODGA)}}_{{i}} ({\text{NO}}_{ 3} )_{3} ]_{\text{org}} }}{{ [ {\text{Am}}^{ 3+ } ][{\text{TODGA}}]_{\text{org}}^{i} [{\text{NO}}_{ 3}^{ - } ]^{3} }}$$

6 $$\beta_{{{\text{L}},i}} = \frac{{ [ {\text{AmL}}_{i}^{3 - 4i} ]}}{{[{\text{Am}}^{ 3+ } ][{\text{L}}^{ 4- } ]^{i} }}$$

7 $$\beta_{{{\text{NO}}_{ 3} ,j}} = \;\frac{{[{\text{Am(NO}}_{3} )_{j}^{3 - j} ]}}{{[{\text{Am}}^{3 + } ][{\text{NO}}_{3}^{ - } ]^{j} }}$$

8 $$K_{{{\text{H}},i}} = \;\frac{{[{\text{H}}_{\text{i}} {\text{L}}^{i - 4} ]}}{{\left[ {{\text{L}}^{4 - } } \right]\left[ {{\text{H}}^{ + } } \right]^{i} }}$$

where *K*_ex_ is the extraction constant, while *β*_L,*i*_ and $\beta_{{{\text{NO}}_{ 3} ,j}}$ denote the apparent stability constants of the americium complexes with the L^4−^ ligand and with nitrate ions, respectively, and *K*_H,*i*_ is the i-th protonation constants of L^4−^. The square brackets denote the molar concentrations of the given species.

The mass balance correlations can be expressed as:

9 $$C_{{{\text{Am}},{\text{tot}}}} = \, \left[ {{\text{Am}}^{ 3+ } } \right] \, + \sum\limits_{j = 1}^{r} {[{\text{Am(NO}}_{3} )_{j}^{3 - j} ]} + \sum\limits_{{i{ = 1}}}^{k} {[{\text{AmL}}_{i}^{3 - 4i} ]} \, + \sum\limits_{{j{ = 1}}}^{s} {[{\text{Am(TODGA)}}_{j} ({\text{NO}}_{3} )_{3} ]_{\text{org}} }$$

10 $$C_{{{\text{L}},{\text{tot}}}} = \, \left[ {{\text{L}}^{{ 4{-}}} } \right]\; + \sum\limits_{{i{ = 1}}}^{k} {i [ {\text{AmL}}_{i}^{3 - 4i} ]} \; + \sum\limits_{i = 1}^{z} {[{\text{LH}}_{i}^{i - 4} ]}$$

where $[{\text{L}}^{ 4- } ]$ denotes the molar concentration of the “free” (unbound, unprotonated) L^4−^ ligand in the aqueous phase, and subscript “tot”—the total concentration of the given species in the two-phase system. Based on our earlier experimental results [18], the model does not account for the extraction of ligand L to the organic phase. Using Eqs. (6), (8) and (10), we obtain:

11 $$C_{{{\text{L}},{\text{tot}}}} = \, \left[ {{\text{L}}^{{ 4{-}}} } \right]\left(1\; + \sum\limits_{i = 1}^{z} {{\text{K}}_{{{\text{H,}}i}} [{\text{H}}^{ + } ]^{i} }\right)\; + [{\text{Am}}^{3 + } ]\sum\limits_{{i{ = 1}}}^{k} {i\beta_{{{\text{L,}}i}} } [{\text{L}}^{4 - } ]^{i}$$

where the last term becomes negligible at a trace Am^3+^ concentration. The distribution ratio of Am^3+^ in the two-phase system studied, *D* = *C*_Am,org_/*C*_Am,aq_, can be expressed as:

12 $$D = \frac{{\sum_{{j{ = 1}}}^{s} {[{\text{Am(TODGA)}}_{j} ({\text{NO}}_{ 3} )_{3} ]_{\text{org}} } }}{{[{\text{Am}}^{3 + } ] + \sum_{{j{ = 1}}}^{\text{w}} {[{\text{Am(NO}}_{3} )_{j}^{3 - j} ] + \sum_{{i{ = 1}}}^{k} {[{\text{AmL}}_{i}^{3 - 4i} ]} } }}$$

where, in the absence of L, we have *D* = *D*_0._

13 $$D_{0} = \frac{{\sum_{{j{ = 1}}}^{s} {[{\text{Am(TODGA)}}_{j} ({\text{NO}}_{ 3} )_{3} ]_{\text{org}} } }}{{[{\text{Am}}^{3 + } ] + \sum_{{j{ = 1}}}^{\text{w}} {[{\text{Am(NO}}_{3} )_{j}^{3 - j} ]} }}$$

The combination of Eqs. (6), (7), (12) and (13) leads to the equation:

14 $$\sum\limits_{{i{ = 1}}}^{k} {\beta_{{{\text{L,}}i}} } [{\text{L}}^{4 - } ]^{i} \; = \;\left( {\frac{{D_{0} }}{D} - 1} \right)\left( {1\; + \;\sum\limits_{{j{ = 1}}}^{\text{w}} {\beta_{{{\text{NO}}_{ 3} ,j}} [{\text{NO}}_{3}^{ - } ]^{j} } } \right)$$

## Experimental

### Materials

The extractant and the hydrophilic ligand studied, TODGA and SO_3_–Ph–BTP, were purchased from Technocomm LTD (UK). TODGA was used as received. The SO_3_–Ph–BTP preparation was additionally purified as described in [18]. Chemical- and analytical-grade kerosene and 1-octanol (both Sigma Aldrich) were used as the diluent and modifier, respectively. The ^241^Am radiotracer, 0.4 MBq mL^−1^ (ca. 13 μM) in 1.0 M HCl containing 0.36 mM La(III) as a carrier, was purchased from POLATOM, Otwock-Świerk (Poland).

### Solvent extraction

Solutions of TODGA and SO_3_–Ph–BTP were prepared from precisely weighed amounts of the reagents. The aqueous phase of a constant ionic strength contained nitric acid (POCH Gliwice) and sodium nitrate (Merck, ACS Reagent) of total concentration in deionized water equal to 1.00 M, and the SO_3_–Ph–BTP ligand in the range: *C*_L,tot_ = 0.032–52.4 mM. 5 μL of the radiotracer solution was added to 0.5 mL of the aqueous phase, so that the specific radioactivity of ^241^Am and the concentration of La^3+^ carrier in the initial aqueous phase were equal to 4 kBq mL^−1^ (ca. 0.13 μM) and 4 μM, respectively. The acidity of the aqueous phase with ^241^Am varied from 0.02 to 1 M HNO_3_. The organic phase consisted of 0.1 M TODGA in 5 vol% octanol–kerosene, except for 1 M HNO_3_ (0.06 M TODGA). Because of significant HNO_3_ extraction to organic solutions of TODGA [20], the organic phase was pre-equilibrated with the aqueous phase containing no SO_3_–Ph–BTP. Solvent extraction experiments were carried out in plastic vials of Eppendorf type. The volumes of the organic and aqueous phase were equal to 0.4 mL each. The vials with the two phases were mechanically shaken at 1400 rpm in the thermomixer for 30 min at 25 ± 0.1 °C to achieve equilibrium (preliminary studies have shown that the *D* values are reproducible when shaking the phases from 15 to 90 min). After shaking, the phases were centrifuged at 7000 rpm for 5 min and separated. Two aliquots of 0.1 mL of each phase were taken for further analysis. The radioactivities of ^241^Am in both phases were measured by gamma spectrophotometry at the energy of 59.5 keV.

## Results and discussion

**Fig. 1:**
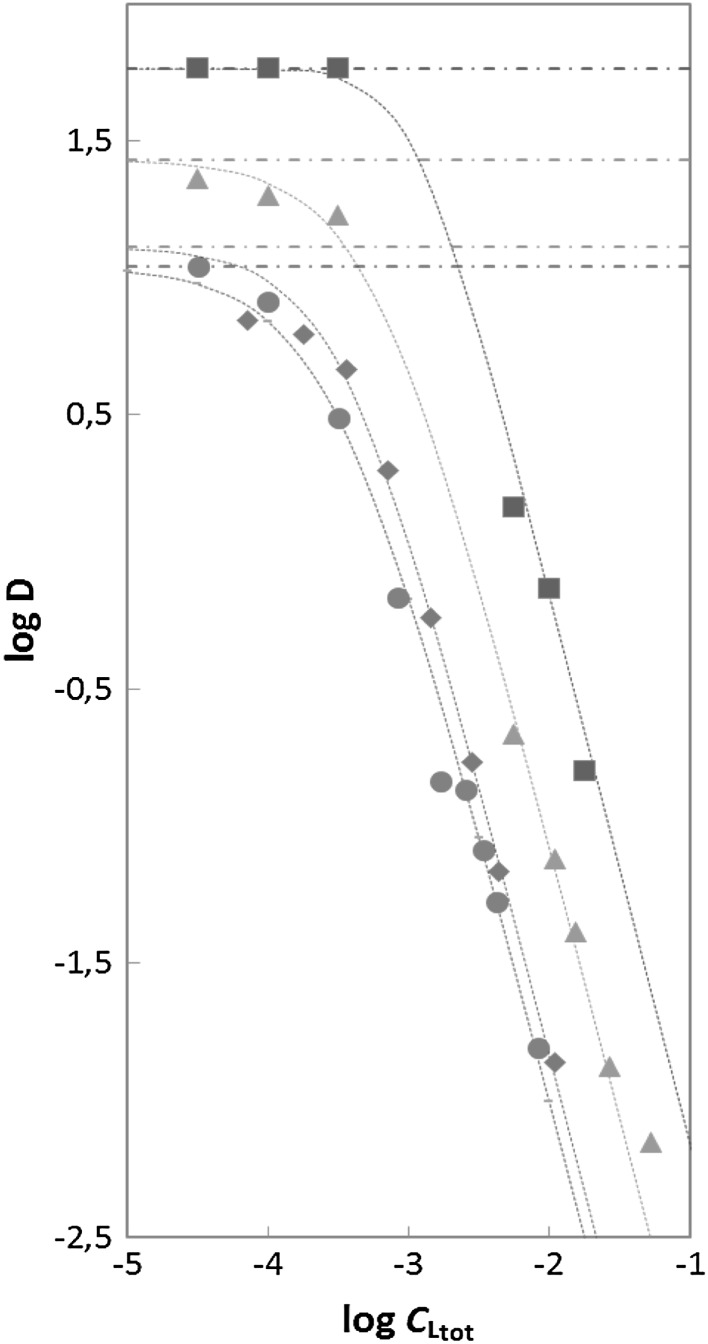
Log–log dependence of the distribution ratio of Am(III), *D*, on the total molar concentration of SO_3_–Ph–BTP in the aqueous phase, *C*_L,tot_, at various acidities: *brown filled square* 1 M; *green filled triangle* 0.5 M; *blue filled diamond* 0.15 M; *red filled circle* 0.02 M HNO_3_, at a constant 1 M nitrate concentration, at 25 °C. The organic phase was 0.1 M TODGA in 5 vol% octanol–kerosene. The *dashed horizontal lines* correspond to the *D*_0_ values at the given acidities

The dependences of log*D* on log*C*_L,tot_ at various acidities of the aqueous phase are shown in Fig. 1.

A bunch of curves is observed in Fig. 1, with different *D*_0_ values increasing with increased the acidity of the aqueous phase: *D*_0_ = 11.0 ± 0.1, 13.0 ± 0.7, 27.0 ± 1.9 and 58.0 ± 1.7 for 0.02, 0.15, 0.5 and 1 M HNO_3_, respectively. The *D* values for 1 M HNO_3_, originally obtained with the 0.06 M TODGA organic phase (with *D*_0_ = 6.0 ± 1.0), have been adjusted to the standard conditions. The competition for Am ions between the lipophilic TODGA and the hydrophilic SO_3_–Ph–BTP^4−^ (L^4−^) ligands leads to the decrease of the *D* values with increasing L concentration. Moreover, the significant increase in the *D* values with increasing HNO_3_ concentration is observed at the same [L]_tot_ values. At higher acidities this effect is significantly greater than that observed in the *D*_0_ values, which points to a significant change in the protonation of L^4−^ in the examined range of acidity (Eqs. 4 and 8). The hydrophilic LH^3−^ ligands (protonated at the donor N atoms of the central pyridine moiety [21]) do not complex the Am^3+^ ions in the aqueous phase.

To conclude on the complex formation of Am^3+^ ions with free ${\text{L}}^{ 4- }$ ligand in the aqueous phase, we applied the known solvent extraction method for determining stability constants of metal complexes with hydrophilic ligands [17], we had used before when studying complexation of ${\text{UO}}_{2}^{2 + }$ ions [18]. The log(*D*_0_/*D* − 1) values were plotted as a function of ${ \log }[{\text{L}}^{ 4- } ]$. In the regions where a given complex (1:1 or 1:2) predominates, Eq. (14) can be simplified and expressed in the logarithmic form:

15 $${ \log }\left( {\frac{{D_{0} }}{D} - 1} \right) + { \log }\left( { 1 { } + \sum\limits_{{j{ = 1}}}^{r} {\beta_{{{\text{NO}}_{ 3} ,j}} [{\text{NO}}_{3}^{ - } ]^{j} } } \right) \, = \, i{ \log }\left[ {{\text{L}}^{{ 4{-}}} } \right] \, + { \log }\beta_{{{\text{L}},i}}$$

or

16 $$F_{i} \;{ = }\;{ \log }\left( {\frac{{D_{0} }}{D} - 1} \right)\; = \, i{ \log }\left[ {{\text{L}}^{{ 4{-}}} } \right] \, + { \log }\alpha_{i}$$

where

17 $$\alpha_{i} = \beta_{i} /( 1 { } + \varSigma \beta_{{{\text{NO}}_{ 3} ,j}} \left[ {{\text{NO}}_{3}^{ - } } \right]^{j} ) \, = {\text{ const}}$$

**Fig. 2:**
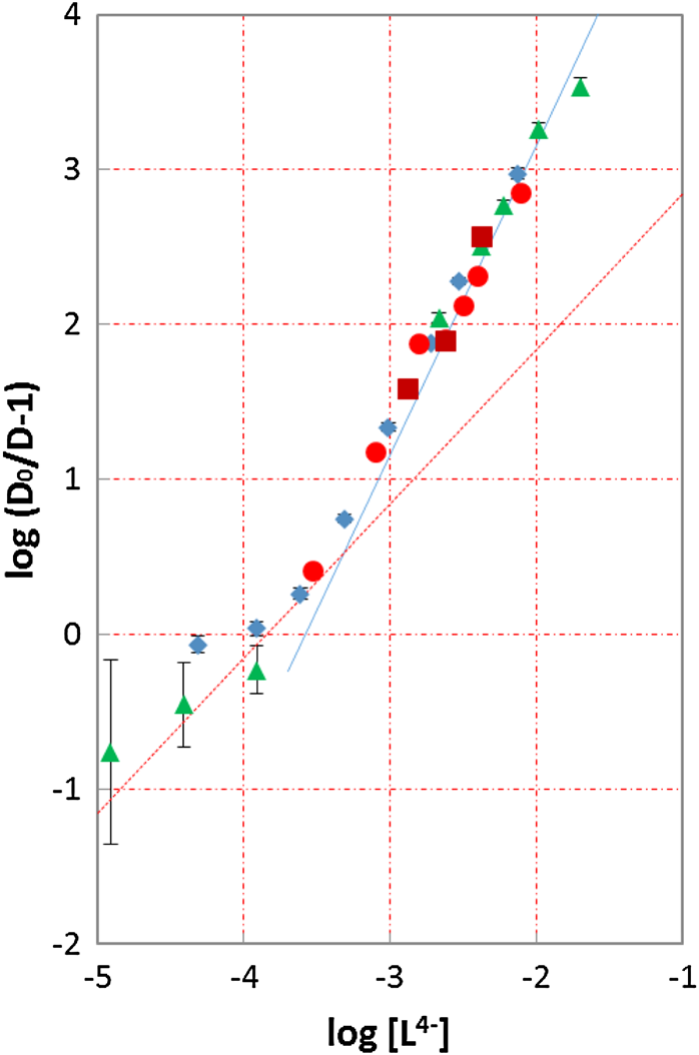
Log(*D*_0_/*D* − 1) for Am^3+^ as a function of ${ \log }[{\text{L}}^{ 4- } ]$ in the system studied at a constant 1 M nitrate concentration and the HNO_3_ concentration equal to: *brown filled square* 1 M; *green filled triangle* 0.5 M; *blue filled diamond* 0.15 M; *red filled circle* 0.02 M, at 25 °C. The ${ \log }[{\text{L}}^{ 4- } ]$ values have been calculated taking log*K*_H,1_ = 0.5. The “best-fit” *straight lines* with the slopes of 1.00 and 2.00 are shown

Extrapolation of the straight lines (16): $F_{i} = f({ \log }\left[ {{\text{L}}^{ 4- } } \right]){\text{ for}}\,i = 1$ and 2 (Fig. 2) to the value ${ \log }\left[ {{\text{L}}^{ 4- } } \right] = 0$ results in obtaining the constant values log*α*_*i*_ from which the stability constants, *β*_*i*_, can be calculated if the $\beta_{{{\text{NO}}_{ 3} ,j}}$ values are known. Following the approach developed recently [18], we calculated (see below) the $\left[ {{\text{L}}^{ 4- } } \right]$ values corresponding to each pair of the experimental variables, *C*_L,tot_ and $[{\text{H}}^{ + } ]$, by finding the optimum log*K*_H,1_ value which ensures the best fit (to the experimental points) of the relationship $F = { \log }\left( {D_{0} /D_{ } - 1} \right) = { \log }(\alpha_{ 1} [{\text{L}}^{ 4- } ] + \alpha_{ 2} [{\text{L}}^{ 4- } ]^{ 2} )$ derived from Eqs. (14) and (17). The fitting was carried out in the whole range of the *C*_L,tot_ and $[{\text{H}}^{ + } ]$ variables, where two consecutive complexes, 1:1 and 1:2, were then found (Fig. 2). Among a dozen values checked up in the range 0 < log*K*_H,1_ < 2 we have found the “best fit” log*K*_H,1_ and then the set of $\left[ {{\text{L}}^{ 4- } } \right]$ values which minimize the sum of weighted (*F*_exp_ − *F*_calc_)^2^ values. The $\left[ {{\text{L}}^{ 4- } } \right]$ values were calculated from Eq. (11) taking *z* = 1, and neglecting the last term because of trace, ca. 10^−7^ M Am^3+^ concentration. Albeit the La^3+^ carrier could also affect the $\left[ {{\text{L}}^{ 4- } } \right]$ values, nonetheless the published log*β*_1_ values for the complexes with lipophilic BTP ligands, much lower for La^3+^ than for Am^3+^ [22], and the low concentration of the ${\text{La}}^{ 3+ }$ carrier, $\left[ {{\text{La}}^{ 3+ } } \right] < 4 \times 10^{ - 6}\, {\text{M}},$ allowed such simplification. The uncertainties were calculated according to the procedure of error propagation of experimental data [23]. The minimum Σ*w*_*i*_(*F*_exp,*i*_ − *F*_calc,*i*_)^2^ (*i* = 1–23) value equal to 0.329 (normalized w_i_) has been obtained at log*K*_H,1_ = 0.5. This “best fit” value is equal to the value determined by Ruff from the UV–Vis spectra of SO_3_–Ph–BTP in aqueous 0–0.9 M HClO_4_ solutions [24].

The plot of log(*D*_0_/*D* − 1) as a function of log[L^4−^] has been shown in Fig. 2. Two regions of linear relationship can clearly be distinguished in the plot, with the slopes of the straight lines equal to one and two. The first region, corresponding to the 1:1 complex, is observed at ${ \log }\left[ {{\text{L}}^{ 4- } } \right] <{-}3. 5$, while the second, corresponding to the 1:2 complex, lies in the range $- 3 < { \log }\left[ {{\text{L}}^{ 4- } } \right] <{-}1. 8$. There is no evidence from the plot for the existence of the 1:3 complex in the aqueous phase, though the limiting concentration of free SO_3_–Ph–BTP^4−^ in the system studied far exceeded 10^−3^ M at which the 1:3 Cm^3+^ complex had been detected with the use of TRLFS method [16].

The values of log(*D*_0_/*D* − 1) calculated by extrapolation of the straight lines with the slopes of 1.00 and 2.00 to the ${ \log }\left[ {{\text{L}}^{ 4- } } \right] = 0$, are equal to log*α*_1_ = 3.844 ± 0.048 and log*α*_2_ = 7.163 ± 0.032, where the uncertainties are equal to two standard deviations. To calculate the stability constants of the 1:1 and 1:2 Am^3+^—SO_3_–Ph–BTP^4−^ complexes using Eq. (17) one must know the $\beta_{{{\text{NO}}_{ 3} ,j}}$ values. Based on the literature review of the stability constants of Am^3+^—NO_3_^*−*^ complexes in aqueous solutions [25] we have estimated these values in 1 M nitrate solution as ${ \log }\beta_{{{\text{NO}}_{ 3} ,1}} = 0. 2 5 \, \pm \, 0.0 5 {\text{ and log}}\beta_{{{\text{NO}}_{ 3} ,2}} = {-}0. 4 \, \pm \, 0. 3,$ therefore ${ \log }( 1 + \varSigma \beta_{{{\text{NO}}_{ 3} ,i}} \left[ {{\text{NO}}_{3}^{ - } } \right]^{i} ) = 0. 5 1 \, \pm \,0.0 5.$ This results in the following values of the stability constants of the 1:1 and 1:2 Am^3+^—SO_3_–Ph–BTP^4−^ complexes in the aqueous phase under study: log*β*_1_ = 4.35 ± 0.07 and log*β*_2_ = 7.67 ± 0.06. These conditional stability constants related to aqueous solutions of ionic strength of 1 M are distinctly lower than those reported by Geist et al. for the analogous curium(III) complexes in very dilute aqueous solutions (log*β*_1_ = 5.4 ± 0.1 and log*β*_2_ = 9.3 ± 0.2 [16]). The different ionic strengths of the solutions do not allow to explain this discrepancy, as well as the small difference between the ionic radii of Am^3+^ and Cm^3+^ [26]. Moreover, the Am^3+^ analogue for the 1:3 Cm^3+^ complex (log*β*_3_ = 12.2 ± 0.3 [16]) has not been found in our solvent extraction system.^1^ A reasonable explanation seems to be a hypothesis that an extractable heteroleptic Am^3+^ complex (with e.g. one SO_3_–Ph–BTP^4−^ and two TODGA ligands) forms in the two-phase system under study. This would strongly affect the complex formation equilibria and make the interpretation of the results more complex. In spite of having a similar hypothesis for uranyl ion in the same extraction system rejected [18], the hypothesis may be true in the present case because the first coordination sphere of Am^3+^ is much larger than that of the ${\text{UO}}_{2}^{2 + }$ ion. The research in this direction has already been started. The resolution of this issue should make possible the conclusion whether the calculated log*β*_L.*i*_ quantities are the genuine stability constants of the Am^3+^—SO_3_–Ph–BTP^4−^ complexes, or rather the apparent auxiliary quantities. These apparent quantities well characterize the behaviour of Am^3+^ ions in the particular liquid–liquid extraction system, but they are probably not the “stability constants” in terms of thermodynamics. If this is the case, the model of the solvent extraction process we have used should be modified to allow us to determine the genuine stability constants.

## Conclusions

The results obtained in the present work confirm the observation that the behaviour of Am^3+^ ions, when stripped from a TODGA-containing organic phase to an acidic aqueous nitrate solution containing a hydrophilic anionic ligand, SO_3_–Ph–BTP^4−^, is not in line with expectations based on the stability constants of Cm^3+^—SO_3_–Ph–BTP^4−^ complexes, found in spectroscopic studies. The conditional stability constants of the Am^3+^ complexes (1:1 and 1:2), determined by means of Am^3+^ distribution in the liquid–liquid extraction system, are distinctly lower than the literature values determined by TRLFS for their Cm^3+^ analogues. Moreover, no evidence has been found for the existence (in the extraction system) of the 1:3 Am^3+^—SO_3_–Ph–BTP^4−^ complex similar to the 1:3 Cm(III) complex detected in an aqueous solution alone. However, the apparent stability constants we have determined well describe the behaviour of Am^3+^ ions in the two-phase solvent extraction system, on the contrary to the genuine constants determined by spectroscopy. A hypothesis has been formulated, aimed at understanding the reason of this discrepancy. If this hypothesis is confirmed, the model of the solvent extraction process in the system containing two competing ligands—lipophilic and hydrophilic—will have to be checked on the presence of extra equilibria, acido basic behaviour of the ligands, etc., which can modify the values of the stability constants.

## Footnotes

1 Seeking to ensure that the 1:3 complexes do not really form in the system studied, we carried out an experiment with increased concentrations of SO_3_-Ph-BTP (0.03–0.1 M), decreased acidity (pH 3), and increased concentration of TODGA (0.6 M; *D*_0_ ≈ 200). Also the specific radioactivity of the aqueous phase was increased tenfold (HCl was evaporated from the sample). Unfortunately, the uncertainties of the measured extremely low distribution ratios (*D* < 5 × 10^−4^) were too high to make the results conclusive.
